# Effect of Impaired Stereoscopic Vision on Large-Scale Resting-State Functional Network Connectivity in Comitant Exotropia Patients

**DOI:** 10.3389/fnins.2022.833937

**Published:** 2022-03-08

**Authors:** Han Jin, Ri-Bo Chen, Yu-Lin Zhong, Ping-Hong Lai, Xin Huang

**Affiliations:** ^1^Department of Ophthalmology, Jiangxi Provincial People’s Hospital, The First Affiliated Hospital of Nanchang Medical College, Nanchang, China; ^2^Department of Radiology, Jiangxi Provincial People’s Hospital, The First Affiliated Hospital of Nanchang Medical College, Nanchang, China

**Keywords:** comitant exotropia, independent component analysis, resting-state networks, functional connectivity, functional network connectivity, fMRI

## Abstract

**Background:**

Comitant exotropia (CE) is a common eye movement disorder, characterized by impaired eye movements and stereoscopic vision. CE patients reportedly exhibit changes in the central nervous system. However, it remains unclear whether large-scale brain network changes occur in CE patients.

**Purpose:**

This study investigated the effects of exotropia and stereoscopic vision dysfunction on large-scale brain networks in CE patients *via* independent component analysis (ICA).

**Methods:**

Twenty-eight CE patients (mean age, 15.80 ± 2.46 years) and 27 healthy controls (HCs; mean age, 16.00 ± 2.68 years; closely matched for age, sex, and education) underwent resting-state magnetic resonance imaging. ICA was applied to extract resting-state networks (RSNs) in both groups. Two-sample’s *t*-tests were conducted to investigate intranetwork functional connectivity (FC) within RSNs and interactions among RSNs between the two groups.

**Results:**

Compared with the HC group, the CE group showed increased intranetwork FC in the bilateral postcentral gyrus of the sensorimotor network (SMN). The CE group also showed decreased intranetwork FC in the right cerebellum_8 of the cerebellum network (CER), the right superior temporal gyrus of the auditory network (AN), and the right middle occipital gyrus of the visual network (VN). Moreover, functional network connectivity (FNC) analysis showed that CER-AN, SMN-VN, SN-DMN, and DMN-VN connections were significantly altered between the two groups.

**Conclusion:**

Comitant exotropia patients had abnormal brain networks related to the CER, SMN, AN, and VN. Our results offer important insights into the neural mechanisms of eye movements and stereoscopic vision dysfunction in CE patients.

## Introduction

Comitant exotropia (CE) is a common eye movement disorder, characterized by ocular deviation and impaired stereoscopic vision function; CE affects 1.0% of all children (Govindan et al., 2005). Impaired stereoscopic vision is an important clinical manifestation of CE (Feng et al., 2015). Binocular vision is an important physiological basis for stereoscopic vision. Symmetrical eye positioning allows considerable visual field overlapping between eyes, which is essential for both binocular and stereoscopic vision (Chopin et al., 2019). CE patients exhibit impaired stereoscopic vision because of anomalous retinal correspondence for the same object in both eyes. The fundamental nature of stereoscopic vision depends on good vision in both eyes, good oculomotor control, and intact cortical mechanisms (Li et al., 2017). Moreover, central nervous system function is important for stereoscopic vision.

Stereoscopic vision is a fundamental aspect of human visual function. The visual cortex has an important role in maintaining stereoscopic vision. Neuroimaging studies have shown that the medial temporal lobe plays important roles in stereoscopic depth processing (Backus et al., 2001; Chen et al., 2020). Moreover, the dorsal visual pathway is involved in stereoscopic depth processing (Goncalves et al., 2015). Ban et al. (2012) demonstrated that the dorsal stream is important for binocular vision fusion, while Zheng et al. (2020) found that the dorsal visual regions showed predominant advantages in decoding the disparities present in three-dimensional images. Furthermore, stereoscopic vision is closely related to gray and white matter structures in the brain. Numerous cortical areas are reportedly involved in binocular disparity processing (Ohzawa et al., 1990; Parker, 2007). Abed Rabbo et al. (2018) describe extensive white matter connections between the visual areas and the lateral geniculate nucleus within stereoscopic visual areas. Prins et al. (2017) found that volumetric decreases in the superior lateral occipital cortices are associated with depth perception in monocularly blind patients. Oishi et al. (2018) demonstrated that stereoscopic depth discrimination is involved in dorsoventral communication through the vertical occipital fasciculus. Gillebert et al. (2015) found that the right posterior inferior temporal cortex and right premotor cortex are responsible for three-dimensional shape processing. The aforementioned studies showed that stereoscopic vision is closely related to specific brain structures and functions. However, the effects of impaired stereoscopic vision on large-scale resting-state functional network connectivity (FNC) have not been explored in CE patients.

The human brain exhibits blood oxygenation level-dependent (BOLD) signals in the resting state. Low-frequency fluctuations (<0.01 Hz) in resting-state BOLD signals reflect spontaneous neural activity and imply spatiotemporal correlations in functional networks (Auer, 2008). Low-frequency fluctuations are closely related to several physiological functions including motor (Biswal et al., 1995), vision (Oke et al., 2010), and higher cognitive function (Kong et al., 2016). The low-frequency fluctuations (<0.01 Hz) in BOLD signals reportedly correspond to functionally relevant resting-state networks (RSNs) (e.g., visual, sensorimotor, auditory, default mode, executive, and salience) (Damoiseaux et al., 2006; Smith et al., 2009). The independent component analysis (ICA) method is a powerful data-driven approach for the identification of independent patterns in multivariate data (van de Ven et al., 2004). Importantly, the ICA method does not require a preset seed point, compared with resting-state functional connectivity-based seed points. Moreover, the ICA method has been successfully used to investigate changes in neurophysiological mechanisms that occur in neurological diseases (Du et al., 2015; Chen et al., 2019). To our knowledge, the effects of impaired stereoscopic vision on large-scale resting-state FNC have not been investigated in CE patients.

This study was performed to investigate how impaired stereoscopic vision influences large-scale resting-state FNC in CE patients. We hypothesized that CE patients might exhibit widespread RSN changes involving VNs and vision-related supporting networks. Our findings might provide new insights into the mechanisms of RSN changes that contribute to impaired stereoscopic vision in CE patients.

## Materials and Methods

### Participants

Twenty-eight CE patients and twenty-seven healthy controls (HCs) were recruited from Jiangxi Provincial People’s Hospital, The First Affiliated Hospital of Nanchang Medical College. The diagnostic criteria of CE patients were as follows: (1) comitant exotropia, exodeviation angles between 15 and 80Δ; (2) without a history of strabismus surgery; (3) participant should have undergone fusional control score, Worth 4-dot test, and Titmus stereopsis test.

The exclusion criteria of CE individuals in the study were as follows: (1) additional ocular-related complications (e.g., cataract, glaucoma, high myopia, or optic neuritis); (2) sensory exotropia, fixed exotropia; and (3) concomitant exotropia was associated with amblyopia.

### Magnetic Resonance Imaging Acquisition

Magnetic resonance imaging (MRI) scanning was performed on a 3-T magnetic resonance scanner (Discovery MR 750W system; GE Healthcare, Milwaukee, WI, United States) with eight-channel head coil. Functional images were obtained by using a gradient-echo-planar imaging sequence. All the subjects were instructed to rest quietly with their eyes closed and relaxed without thinking about anything in particular or falling asleep. Whole-brain T1 weights were obtained with three-dimensional brain volume imaging (3D-BRAVO) MRI with the following parameters: repetition time [TR]/echo time [TE] = 8.5/3.3, thickness = 1.0 mm, no intersection gap, acquisition matrix = 256 × 256, field of view = 240 mm × 240 mm, and flip angle = 12°. Functional images were obtained by using a gradient-echo-planar imaging sequence with the following parameters: TR/TE = 2,000 ms/25 ms, thickness = 3.0 mm, gap = 1.2 mm, acquisition matrix = 64 × 64, flip angle = 90°, field of view = 240 mm × 240 mm, voxel size = 3.6 mm × 3.6 mm × 3.6 mm, and 35 axial slices.

### fMRI Data Analysis

All pre-processing was performed using the toolbox for Data Processing and Analysis of Brain Imaging (DPABI^1^) (Yan et al., 2016), which is based on Statistical Parametric Mapping (SPM12)^2^ implemented in MATLAB 2013a (MathWorks, Natick, MA, United States) and briefly the following steps: (1) Remove the first 10 volumes. (2) Slice timing effects, motion corrected. For head motion parameters, more than 2 mm or for whom rotation exceeded 1.5° during scanning were excluded (Van Dijk et al., 2012). (3) Normalized data [in Montreal Neurological Institute (MNI) 152 space] were re-sliced at a resolution of 3 mm × 3 mm × 3 mm. (4) Spatial smoothing by convolution with an isotropic Gaussian kernel of 6 mm × 6 mm × 6 mm full width at half maximum.

### Group Independent Component Analysis

Group ICA was performed to decompose the data into independent components (ICs) using the GIFT toolbox (version 3.0b).^3^ First, 31 IC maps were estimated in this study using the minimum description length criterion to adjust for spatial correlation. Second, the ICs for each subject were derived from the group ICA back-reconstruction step and were converted into *z*-scores (Zuo et al., 2010). Components retained for further analysis among the 31 estimated ICs were selected based on the largest spatial correlation with specific RSN templates (Shirer et al., 2012; Wang et al., 2014). The IC time courses and spatial maps for each subject were transformed to *z*-scores. Fourteen RSNs were identified in this study.

### Intranetwork Functional Connectivity Analysis

The intranetwork FC was represented by the *z*−score of each voxel, which reflects the degree to which the time series of a given voxel correlates with the mean time series of its corresponding component.

### Functional Network Connectivity Analysis

The FNC analysis was performed using the MANCOVAN toolbox in GIFT software to explore changes in the predefined 14 spatial IC pairs of functional connections. First, at 0.01–0.15 Hz, de-trend, de-peak, and low-pass filtering were performed on the selected IC. Then, the pair correlations of these ICs were calculated and transformed using Fisher’s *Z*-transform.

### Statistical Analysis

#### Spatial Maps for Each of the Resting-State Networks

The ICs corresponding to fourteen RSNs were extracted from all subjects. Fourteen of these components coincided with RSNs, namely, IC1 (LECN, left executive control network); IC5 (DAN, dorsal attention network); IC6 (CER, cerebellum network); IC8 (RECN, right executive control network); IC12 (VN1, visual network1); IC13 (DMN1, default mode network1); IC18 (SN, salience network); IC22 (SMN, sensorimotor network); IC23 (AN, auditory network); IC24 (VN2, visual network2); IC26 (VN3, visual network3); IC27 (DMN2, default mode network2); IC29 (VN4, visual network4); and IC30 (DMN3, default mode network3).

#### Intranetwork Functional Connectivity Analysis

Two-sample’s *t*-tests were used to compare differences between the two groups in the intranetwork FC within RSN maps; the Gaussian random field method was used to correct for multiple comparisons and regressed covariates of age and sex using SPM12 software. Group comparisons were masked to the voxels within corresponding RSNs (two-tailed, voxel-level *p* < 0.01, Gaussian random field correction, cluster-level *p* < 0.05).

#### Internetwork Functional Connectivity Analysis

Internetwork functional connectivity analysis was used to calculate temporal relationships between RSNs. Corresponding to the significant correlation combinations, the average time lags were calculated for each group; these represented the amount of delay between time courses of two correlated RSNs. Two-sample’s *t*-tests were used to compare distinct temporal relationships between RSNs between the two groups (*p* < 0.05, uncorrected).

## Results

### Demographics and Visual Measurements

There were no significant differences in the gender and age between the groups (Table 1).

**TABLE 1:** Demographics and visual measurements between two groups.

Condition	CE group	HC group	*T*-values	*P*-values
Gender (male/female)	(15/13)	(15/12)	N/A	N/A
Comitant category	Congenital exotropia	N/A	N/A	N/A
Age (years)	15.80 ± 2.46	16.00 ± 2.68	−0.240	0.812
Handedness	28 R	27 R	N/A	N/A

*Independent t-test for the other normally distributed continuous data (means ± SD).*

*CE, comitant exotropia; HC, healthy control.*

### Identifications of the Resting-State Networks

**FIGURE 1:**
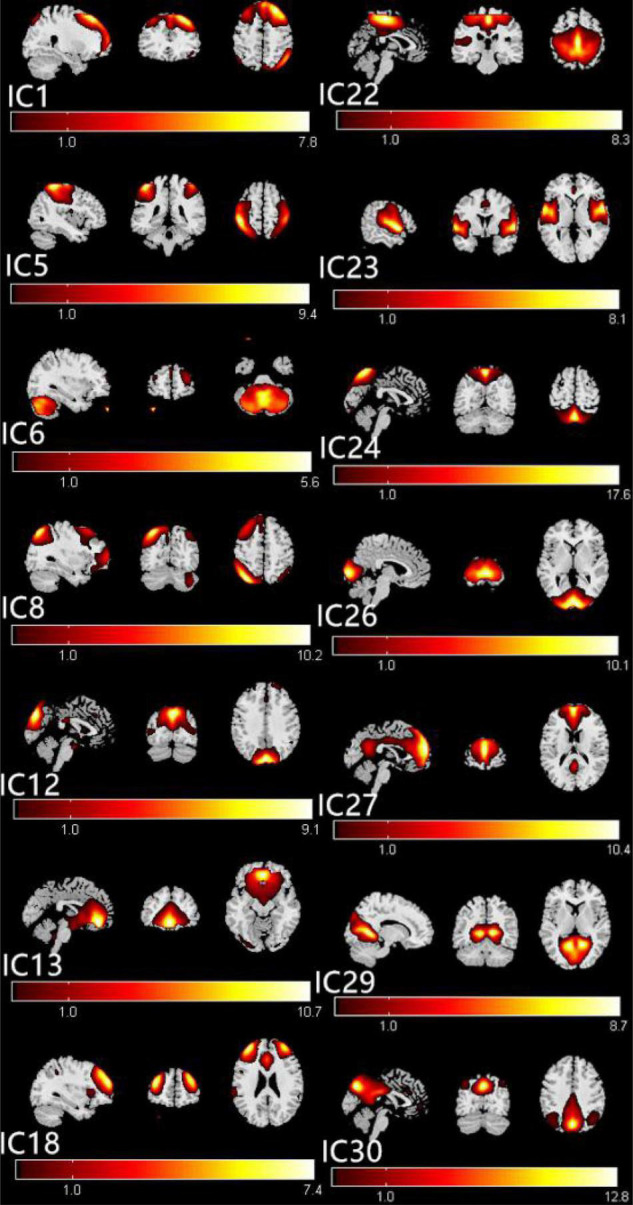
The typical spatial patterns in each RSN of both CE and HC groups, including IC1(LECN), IC5(DAN), IC6(CER), IC8(RECN), IC12(VN1), C13(DMN1), IC18(SN), IC22(SMN), IC23(AN), IC24(VN2), IC26(VN3), IC27(DMN2), IC29(VN4), and IC30 (DMN3). Scale represents *T*-values with a range of 1–17.6 in each RSN. CE, comitant exotropia; HC, healthy control; lECN, left executive control network; DAN, dorsal attention network; CER, cerebellum network; RECN, right executive control network; VN, visual network; DMN, default mode network; SN, salience network; SMN, sensorimotor network; AN, auditory network.

The group ICA approach automatically generated 31 ICs. The typical spatial patterns in each RSN of both CE and HC groups are illustrated in Figure 1. Fourteen of these components coincided with RSNs, namely, IC1 (LECN, left executive control network); IC5 (DAN, dorsal attention network); IC6 (CER, cerebellum network); IC8 (RECN, right executive control network); IC12 (VN1, visual network1); C13 (DMN1, default mode network1); IC18 (SN, salience network); IC22 (SMN, sensorimotor network); IC23 (AN, auditory network); IC24 (VN2, visual network2); IC26 (VN3, visual network3); IC27 (DMN2, default mode network2); IC29 (VN4, visual network4); and IC30 (DMN3, default mode network3).

### Abnormal Intranetwork Functional Connectivity Changes Between Groups

**FIGURE 2:**
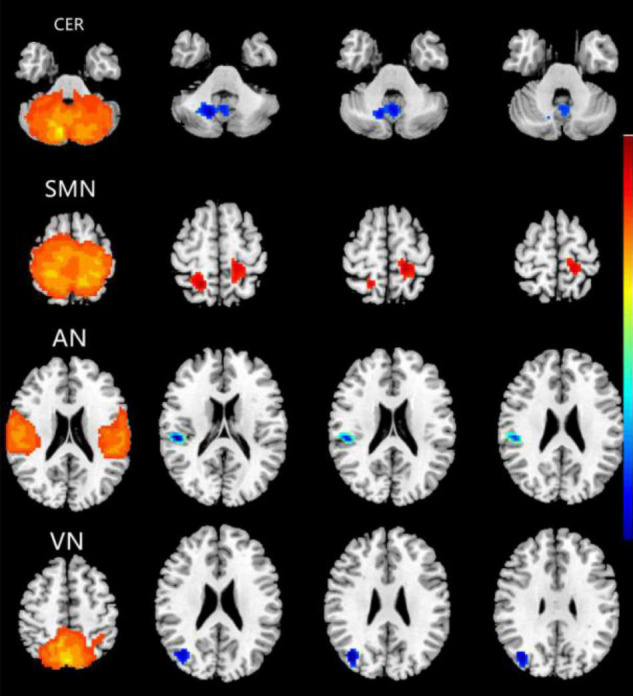
Brain regions with significant differences for five RSNs in the CE group vs. the HC group (two-tailed, voxel-level *p* < 0.01, GRF correction, cluster-level *p* < 0.05). Compared with the HC group, cool colors indicated the decreased functional connectivity and hot colors indicated the increased functional connectivity in the CE group. The pictures correspond to different resting-state networks. CER, SMN, AN and VN. CE, comitant exotropia; HC, healthy control; CER, cerebellum network; SMN, sensorimotor network; AN, auditory network; VN, visual network.

Compared with the HC group, the CE group showed increased intranetwork FC in the bilateral postcentral gyrus of the SMN. Meanwhile, the PD group showed decreased intranetwork FC in the right cerebelum_8 of the CER, the right superior temporal gyrus of the AN, and the right middle occipital gyrus of the VN (Figure 2 and Table 2) (two-tailed, voxel-level *p* < 0.01, GRF correction, cluster-level *p* < 0.05).

**TABLE 2:** Different intranetwork FC of RSNs between two groups.

Condition	RSN	Brain regions	BA	Peak *T*-scores	MNI coordinates (*x*, *y*, *z*)	Cluster size (voxels)
CE < HC	CER	Right Cerebelum_8	–	−4.6045	15, −63, −39	177
CE > HC	SMN	Bilateral postcentral gyrus	–	4.2861	24, −51, 54	152
CE < HC	AN	Right Superior Temporal Gyrus	40	−4.6977	51, −27, 18	96
CE < HC	VN	Right Middle Occipital Gyrus	19	−4.3329	39, −75, 27	88

*The statistical threshold was set at the voxel level with p < 0.01 for multiple comparisons using Gaussian random-field theory (voxel-level p < 0.01, GRF correction, cluster-level p < 0.05). T-score represents the statistical value of peak voxel showing the differences in FC between the two groups.*

*CE, comitant exotropia; HC, healthy control; FC, functional connectivity; RSNs, resting-state networks; BA, Brodmann area; MNI, Montreal Neurologic Institute; CER, cerebellum network; SMN, sensorimotor network; AN, auditory network; VN, visual network.*

### Functional Network Connectivity Analysis

**FIGURE 3:**
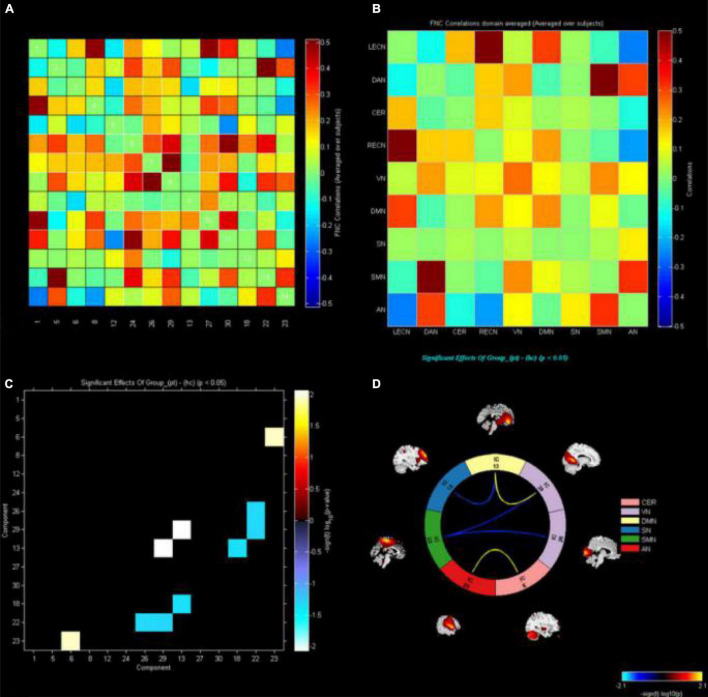
The functional connectivity correlation matrix between all RSNs. The color scale represents the value of the correlations. Warm color represents positive correlations, and cold color represents anti–correlations. The markers indicate significant functional connection between networks IC1(LECN), IC5(DAN), IC6(CER), IC8(RECN), IC12(VN1), C13(DMN1), IC18(SN), IC22(SMN), IC23(AN), IC24(VN2), IC26(VN3), IC27(DMN2), IC29(VN4), and IC30 (DMN3) (*p* < 0.05 uncorrected). **(A,B)** Matrix shows differences of internetwork functional connectivity between two groups. **(C)** Significance and direction following two–sample’s *t*–tests (CE–HC) on each pairwise correlation are depicted as the –sign (*t* value) log10 (*p* value); the CER-AN, SMN-VN, SN-DMN, and DMN-VN connections were found to be significantly altered between two groups (*p* < 0.05). **(D)**. CE, comitant exotropia; HC, healthy control; lECN, left executive control network; DAN, dorsal attention network; CER, cerebellum network; RECN, right executive control network; VN, visual network; DMN, default mode network; SN, salience network; SMN, sensorimotor network; AN, auditory network.

The markers indicate significant functional connection between networks IC1 (LECN), IC5 (DAN), IC6 (CER), IC8 (RECN), IC12 (VN1), C13 (DMN1), IC18 (SN), IC22 (SMN), IC23 (AN), IC24 (VN2), IC26 (VN3), IC27 (DMN2), IC29 (VN4), and IC30 (DMN3) (*p* < 0.05, uncorrected). Matrix shows differences of internetwork functional connectivity between two groups (Figures 3A,B). Significance and direction following two-sample *t*-tests (CE-HC) on each pairwise correlation are depicted as the −sign(*t* val)log10(*p* val) (Figure 3C); the CER-AN, SMN-VN, SN-DMN, and DMN-VN connections were found to be significantly altered between two groups (*p* < 0.05) (Figure 3D).

## Discussion

To our knowledge, this is the first study to investigate the effects of impaired stereoscopic vision on large-scale resting-state FNC in CE patients using the ICA method. Compared with the HC group, the CE group showed increased intranetwork FC in the bilateral postcentral gyrus of the SMN; it also showed decreased intranetwork FC in the right cerebellum_8 of the CER, the right superior temporal gyrus of the AN, and the right middle occipital gyrus of the VN. Moreover, CER-AN, SMN-VN, SN-DMN, and DMN-VN connections were significantly altered between the two groups.

### Altered Intranetwork Functional Connectivity in the Comitant Exotropia Group

The CE group showed decreased intranetwork FC in the right cerebellum_8 of the CER. The CER is closely related to movement and balance functions. Moreover, the cerebellum is important for controlling eye movements and binocular disparity (Gulyas and Roland, 1994; Versino et al., 1996; Beh et al., 2017). Ouyang et al. (2017) reported that comitant strabismus patients showed changes in gray matter volume in the left cerebellum. Musazadeh et al. (2004) reported that cerebellar tumors caused esotropia. Leguire et al. (2011) demonstrated that patients with idiopathic infantile nystagmus syndrome exhibited significant activation increases in the declive of the cerebellum. Consistent with these prior reports, we found that the CE group had decreased functional connectivity in the right cerebellum_8 of the CER, which might lead to impaired eye movement and binocular vision in CE patients.

Notably, the CE group exhibited increased intranetwork FC in the bilateral postcentral gyrus of the SMN. The postcentral gyrus (i.e., the sensorimotor gyrus) has an important role in proprioception, which implies involvement in providing sensory information that helps to control voluntary movement. Sweeney et al. (2007) demonstrated that sensorimotor brain systems have critical roles in eye movement control, while van Beers et al. (2001) reported that sensorimotor integration is involved in the localization of static objects during smooth pursuit eye movements. Sensorimotor function is also closely related to the formation of stereovision (Ringach et al., 1996). Here, we found that CE patients showed decreased functional connectivity within the SMN, which might lead to the impaired eye movements and stereoscopic vision observed in such patients.

The AN is located in the temporal lobe, which is involved in auditory information processing. The CE group showed decreased intranetwork FC in the right superior temporal gyrus of the AN. There are close relationships between the visual and auditory systems; cross-modal interactions between these systems have been observed in patients with vision loss (Collignon et al., 2007; Lunghi et al., 2014). Petrus et al. (2015) demonstrated that vision loss contributes to cross-modal interactions between the primary auditory and visual cortices. To our knowledge, no studies have shown that the loss of stereovision might contribute to auditory dysfunction. In the present study, the CE group showed decreased functional connectivity within the AN, which might lead to the loss of stereoscopic vision and thus contribute to auditory dysfunction. In the future, we plan to investigate the underlying neural mechanism of AN dysfunction in CE patients.

Furthermore, we found that the CE group showed decreased intranetwork FC in the right middle occipital gyrus of the VN. The VN is located in the occipital cortex, which is involved in visual information processing. Wong et al. (2005) revealed metabolic suppression in the visual cortices of strabismic macaques (Economides et al., 2021). Additionally, Yan et al. (2019) reported that CE patients had increased functional connectivity between the primary visual cortex and other brain areas. Yang et al. (2014) found that the infantile esotropia group showed lower BOLD signals in the left cingulate gyrus, bilateral precuneus, and left angular gyrus during a visual stimulus, compared with controls. Yan et al. (2010) reported that adult strabismus patients had decreased white matter volumes in the right middle occipital gyrus and right occipital lobe/cuneus. Consistent with these prior reports, we also found that the CE group showed decreased functional connectivity within the VN.

Finally, FNC analysis showed that CER-AN, SMN-VN, SN-DMN, and DMN-VN connections were significantly altered between the two groups. We speculate that altered interactions among these networks might lead to RSN compensation in CE patients with stereoscopic vision loss.

Some limitations should be acknowledged in this study. First, the sample size of CE patients in our study was small. Second, RSN values based on fMRI signals would still be affected by physiological noise, such as cardiac and respiratory activity.

## Conclusion

Comitant exotropia patients had abnormal brain networks related to the CER, SMN, AN, and VN. Our results offer important insights into the neural mechanisms of eye movements and stereoscopic vision dysfunction in CE patients.

## Data Availability Statement

The raw data supporting the conclusions of this article will be made available by the authors, without undue reservation.

## Ethics Statement

The studies involving human participants were reviewed and approved by the Medical Ethics Committee of the Jiangxi Provincial People’s Hospital Affiliated to Nanchang University. Written informed consent to participate in this study was provided by the participants’ legal guardian/next of kin.

## Author Contributions

HJ, R-BC, Y-LZ, P-HL, and XH contributed to data collection and statistical analyses and wrote the manuscript and designed the protocol, and contributed to the MRI analysis. HJ, R-BC, and Y-LZ designed the study and oversaw all clinical aspects of study conduct and manuscript preparation. All authors contributed to the article and approved the submitted version.

## Conflict of Interest

The authors declare that the research was conducted in the absence of any commercial or financial relationships that could be construed as a potential conflict of interest.

## Publisher’s Note

All claims expressed in this article are solely those of the authors and do not necessarily represent those of their affiliated organizations, or those of the publisher, the editors and the reviewers. Any product that may be evaluated in this article, or claim that may be made by its manufacturer, is not guaranteed or endorsed by the publisher.

## Footnotes

1
http://www.rfmri.org/dpabi

2
http://www.fil.ion.ucl.ac.uk

3
http://icatb.sourceforge.net/
